# F7 and topotecan co-loaded thermosensitive liposome as a nano-drug delivery system for tumor hyperthermia 

**DOI:** 10.1080/10717544.2020.1772409

**Published:** 2020-06-08

**Authors:** Chunyang Du, Shuangshuang Li, Yuan Li, Hervé Galons, Na Guo, Yuou Teng, Yongmin Zhang, Mingyuan Li, Peng Yu

**Affiliations:** a College of Biotechnology, China International Science and Technology, Cooperation Base of Food Nutrition/Safety and Medicinal Chemistry, Tianjin International Cooperation Research Centre of Food Nutrition/Safety and Medicinal Chemistry, Tianjin University of Science & Technology/Tianjin Enterprise Key Laboratory for Application Research of Hyaluronic Acid, Tianjin, China; b Institut Parisien de Chimie Moléculaire, UMR CNRS 8232, Paris, France

**Keywords:** F7, topotecan, thermosensitive liposome, tumor hyperthermia, co-delivery

## Abstract

In order to enhance the targeting efficiency and reduce anti-tumor drug’s side effects, topotecan (TPT) and F7 were co-loaded in thermosensitive liposomes (F7-TPT-TSL), which show enhanced permeability and retention in tumors, as well as local controlled release by heating *in vitro*. TPT is a water-soluble inhibitor of topoisomerase I that is converted to an inactive carboxylate structure under physiological conditions (pH 7.4). F7 is a novel drug significantly resistant to cyclin-dependent kinase but its use was restricted by its high toxicity. F7-TPT-TSL had excellent particle distribution (about 103 nm), high entrapment efficiency (>95%), obvious thermosensitive property, and good stability. Confocal microscopy demonstrated specific higher accumulation of TSL in tumor cells. MTT proved F7-TPT-TSL/H had strongest cell lethality compared with other formulations. Then therapeutic efficacy revealed synergism of TPT and F7 co-loaded in TSL, together with hyperthermia. Therefore, the F7-TPT-TSL may serve as a promising system for temperature triggered cancer treatment.

## 1. Introduction

Malignant tumors are among the most serious diseases affecting human health worldwide. Chemotherapy remains the main anti-tumor strategy; however, the treatment results are often unsatisfactory (Visser et al., 2018; Lange et al., 2019) because of the severe dose-limiting side effects and development of drug resistance after multiple administrations of chemotherapy (Robak et al., 2018; Russo & Sundaramurthi, 2019). Additionally, chemotherapy drugs are not selective for tumor tissues, often causing different degrees of damage to normal cells while killing tumor cells (Bergerot et al., 2019). To improve therapeutic efficacy, multidrug approaches are particularly valuable.

Nano drug carriers have made great achievements, and exogenous carriers are currently studied mostly such as liposomes (Jhaveri et al., 2018), nanoparticles (Ashfaq et al., 2017), microspheres (albumin microspheres, gelatin microspheres, ethylcellulose microspheres, starch microspheres) (Thakare et al., 2017; Nouri et al., 2019) and dendrimer (Bello et al., 2017). In addition, endogenous drug carriers such as cell membranes and exosomes are gradually being studied (Reyes-Ruiz et al., 2019; Su et al., 2020). Using nanodelivery systems, the co-delivery of multiple drugs can increase drug bioavailability and reduce their nonspecific toxicity. Liposomes have good biocompatibility and biodegradability and low toxicity (Morgan et al., 1985; Mu et al., 2017; Johnsen et al., 2018). The liposome surface can be coated with inert polymer molecules such as oligosaccharides, glycoproteins, polysaccharides, and synthetic polymers to prepare ‘invisible’ liposomes, extending their half-lives (Zhang et al., 2016; Li et al., 2017). Using a controlled process, liposomes can be prepared that encapsulate both fat-soluble and water-soluble drugs in particles with sizes of 100–150 nm. Using these liposomes, phagocytosis of the reticuloendothelial system can be avoided in the blood circulation to reduce toxicity (Jain & Jain, 2016). Based on the tumor enhanced permeability and retention effect, liposomes can accumulate at the tumor site to improve drug efficacy (Tiet & Berlin, 2017; Golombek et al., 2018; Li et al., 2019). However, the release of drugs from liposomes is relatively slow; therefore, various response triggers have been investigated to improve drug release, including pH (Yuba et al., 2018), temperature (Lokerse et al., 2016), ultrasound (Wang et al., 2018), photodynamic conversion (Lee et al., 2018), and microwaves (Jin et al., 2016), which allow drugs to be rapidly and specifically released at the tumor site. In 1985, hyperthermia was certified by the FDA as the fifth most widely used cancer treatment method after surgery, radiation therapy, chemotherapy, and biotherapy (Shetake et al., 2016; Mitxelena-Iribarren et al., 2020). Tumor hyperthermia kills tumor cells by heating the tumor site to a temperature slightly above body temperature (Shaterabadi et al., 2018; Das et al., 2019).

Thermosensitive liposomes (TSLs) have been used as targeted drug carriers in combination with tumor hyperthermia to achieve synergistic effects (Ta & Porter, 2013; Ta et al., 2014): (1) the drug can be highly enriched in tumor tissue. After local heating of the tumor, the membrane fluidity of the cancer cells and vascular permeability of the tumor increase, and thus hyperthermia can promote entry of the drug into tumor cells. The phase of the TSL bilayer film changes to a liquid crystal state with high fluidity and the film structure loosens so that the packaged product is released quickly (Ta et al., 2010; Evans, 2017). (2) Many chemotherapeutic drugs induce apoptosis through different mechanisms, which can be promoted by hyperthermia. (3) Hyperthermia can reach a higher temperature in the central part of tumor tissues, an acidic environment where tumor cell apoptosis is likely to occur (Haemmerich & Motamarry, 2018). Heating can improve blood circulation around the tumor, which is beneficial for drug entry (Gao et al., 2019). Additionally, drug release induced by increased temperature (approximately 42 °C) has been confirmed *in vitro* and mice. Various preclinical experiments performed to analyze hyperthermia combined with TSLs have shown promising results, such as ThermoDox in III clinical experiments (Yatvin et al., 1978; Poon & Borys, 2009; Hashemi et al., 2018).

Topotecan (TPT) is a water-soluble semi-synthetic camptothecin derivative that inhibits topoisomerase I and specifically interacts with Topo1-DNA to form a ‘drug-Top1-DNA’ ternary complex (Tsuda & Kitamasu, 2019). It inhibits DNA single-strand breakage and rejoining, prevents DNA replication, and leads to apoptosis (Pommier, 2006; Padhi et al., 2018). In 1996, TPT was approved by the FDA for treating ovarian cancer and small cell lung cancer. TPT has broad-spectrum anti-tumor spectrum effects and strongly inhibits various cancer cell lines, indicating its good potential for clinical application. However, TPT is unstable under physiological conditions because the ring-opening of the drugs at physiological pH (pH 7.4) results in conversion to an inactive carboxylate structure, affecting drug efficacy (Rosca et al., 2015; Xing et al., 2015; Xu & Pan, 2019). To contribute to the removal of the lactone ring-opening, Tarhan et al. prepared magnetic dextran branched with NαNα-Bis (carboxymethyl)-l-lysine hydrate (NTA) nanoparticles (MD 3) to release drug at the pH of tumor environment (Tarhan et al., 2019).

The novel purine derivative F7, synthesized by Professor Herve Galons’ team at the Institute of Pharmaceutical Sciences of the French Academy of Sciences, is a promising anticancer drug that acts on the pathological state of imbalance between apoptosis and cell division and has broad-spectrum anti-tumor effects. F7 has shown curative effects in lung cancer, breast cancer, and liver cancer, among other cancer types, including excellent effects against chronic lymphocytic leukemia and multiple cysts. It is significantly resistant to cyclin-dependent kinase compared to roscovitine (Hobbs et al., 1998; Ott et al., 2017). However, in pharmacodynamics research *in vivo*, the systemic toxicity of F7 in mice was shown to be severe, limiting the drug development of F7. Therefore, it may be necessary to combine F7 drugs with other drugs to reduce dose and side effects while avoiding drug resistance.

In this study, we designed and developed a co-loaded thermosensitive liposome (F7-TPT-TSL) which was simultaneously loaded with F7 and TPT by the pH gradient method to stabilize the drug in the inner aqueous phase. These particles had suitable sizes and thermal properties for tumor treatment applications. After tail vein injection to xenograft tumor model nude mice, the co-loaded thermosensitive liposomes first accumulated at the tumor site through blood circulation; upon heating to 42.5 °C, the drug was rapidly released and F7-TPT-TSL showed reduced toxicity, high effectiveness, and anti-drug effects.
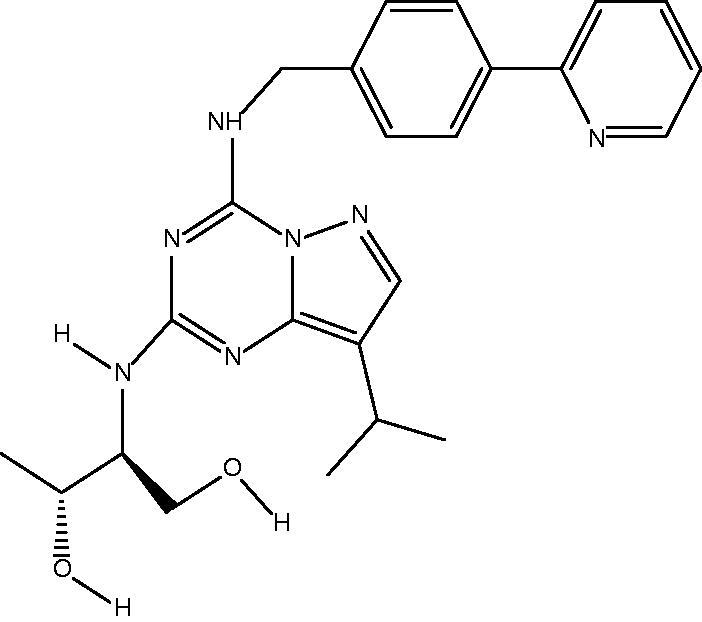


## 2. Materials and methods

### 2.1. Materials

Compound F7 was synthesized by the Galons team of the Institute of Pharmaceutical Sciences of the French Academy of Sciences; Topotecan was from Meryer Chemical Technology Co., Ltd. (Shanghai, China); 1,2-dipalmitoyl-sn-glycero-3-phosphocholine (DPPC); 1,2-distearoyl-sn-glycero-3-phosphoethanolamine-N-(amino (polyethylene glycol)-2000) (DSPE-mPEG_2000_), and myristoyl hemolytic lecithin (MSPC) were purchased from A.V.T. (Shanghai, China); methanol (chromatographic pure grade) was acquired from Concord Co., Ltd. (Tianjin, China); CHCl_3_ was from Sinopharm Chemical Reagent Co., Ltd. (Shanghai, China); RPMI-1640 was a gift from Sigma-Aldrich (Steinheim, Germany); Milli-Q water was used in all the experiments.

### 2.2. Preparation of different liposome formulations

F7 and TPT were simultaneously loaded into liposomes, formulated with DPPC:DSPE-PEG_2000_:MSPC = 81:16:3 (weight ratio) using the active loading way (Li et al., 2016). First of all, the blank liposomes were prepared by lipid film hydration and extrusion. The lipids were dissolved in chloroform and the solvent was removed under vacuum in rotary evaporator forming the homogeneous lipid film, vacuum dried for 8 h. Then lipid film was hydrated in 200 mM citric acid buffer solution at 50 °C. The newly formed multilamellar lipid vesicles were shattered by ultrasound, extruding through a 200 nm polycarbonate filter and resulted in small TSL with a uniform size.

F7 and TPT (1:1, w/w) were loaded into the liposome using the pH gradient method at 1:30 drug/lipids mass ratio. Liposome suspension was directly added into 200 mM citric acid buffer drug solution, and then we adjusted the pH of the extrinsic phase to 7.5 using Na_2_CO_3_ solution. As a result, liposomes with a pH gradient between extrinsic and interior phase were developed, then incubated for about 30 min. In the end, most drug molecules were locked in the interior phases as ionotropic F7 and TPT. Then, F7-TPT-TSL was sterilized by 200 nm polycarbonate filter and subpackaged to aseptic pails.

F7 thermosensitive liposomes (F7-TSL) and topotecan thermosensitive liposomes (TPT-TSL) were prepared as described above.

### 2.3. Dynamic light scattering

Particle size, polydispersity index (PDI), and zeta potential of different liposome formulations were measured by dynamic light scattering (DLS) technique via Zetasizer NanoZS90 (Nano-ZS90, Malvern Instrument, Malvern, UK) at 25 ± 1 °C.

### 2.4. Transmission electron microscope (TEM)

The TEM imaging was performed on transmission electron microscopy (JEM-1010, JEOL Ltd., Tokyo, Japan) operated at an acceleration voltage of 80 kV. For sample preparation, 20 μL of F7-TPT-TSL were dropped onto 300 mesh copper TEM grid and the excess dispersions were then removed after 3 min; after that, the adsorbed sample was stained with 20 μL of 3% phosphotungstic acid for 30 s.

### 2.5. Differential scanning calorimetry

Differential scanning calorimetry (DSC) thermograms were obtained by loading the samples into an aluminum pan and subjected to the heat cycle in a differential scanning calorimeter (DSC 200 F3 Maia, NETZSCH, Selb, Germany) at a ramp rate of 10 °C/min from 20 to 80 °C. All the cycles were operated under a nitrogen gas atmosphere. An empty pan was employed as a reference to eliminate the calorimetric effect of the pan.

### 2.6. Entrapment efficiency

HPLC was used for the quantitative determination of F7 and TPT. The separation was performed on a column (Eclipse XDB-C18 4.6 mm × 150 mm, 5 μm). The mobile phase: methanol 25–50%, 0–10 min; 50–75%, 10–30 min; 1.5% triethylamine aqueous solution (adjusted with phosphoric acid to pH 4.0) 75–50%, 0–10 min; 50–75%, 10–30 min. Mobile phase was pumped at a flow rate of 1.0 mL/min and the detection wavelength was 268 nm.

An ultrafiltration technique was used to separate the unencapsulated F7 and TPT from liposomes. F7-TPT-TSL was diluted 10 times with Milli-Q water and then placed in the upper chamber of a centrifuge tube matched with an ultrafilter (Sartorius Vivaspin 500 μL, 30 k MWCO PES, Goettingen, Germany) and was centrifuged for 10 min at 11,180×*g*. The ultrafiltrate in the ultrafilter containing the unencapsulated drug was determined by HPLC, as described above. The total drug in F7-TPT-TSL was determined through SDS solution (5%) disruption by HPLC. The entrapment efficiency (EE%) was calculated using the following equation:

(1) $$\mathrm{EE}\;(\%)=(1-\frac{C_{x}}{C_{y}})100\%$$

EE (%) was obtained according to Equation (1), where *C_y_* and *C_x_* represent the total drug in TSL and the amount of free drug in the ultrafiltrate, respectively.

### 2.7. Stability study

After the blank-TSL and F7-TPT-TSL were stored at 4 °C for 1 month, their stability in different 10-fold dilutions was tested. The stability was monitored with a Turbiscan Lab^®^ Expert (Formulaction, Toulouse, France), an innovative analytical instruments to determine the small changes of colloidal systems. To obtain these data, the samples were placed into cylindrical glass tubes and then submitted to Turbiscan Lab^®^ Expert at predesigned time points. The stability was evaluated with delta transmission and delta backscattering as indexes.

### 2.8. *In vitro* release kinetics

Release of F7 and TPT from F7-TPT-TSL as a function of temperature (37 °C and 41 °C) was performed using a dialysis technique. In this study, liposomes with a volume of 2 mL were added into a cellulose acetate dialysis bag (MWCO 8–14 kDa) and immersed in 68 mL PBS buffer medium (pH7.4, containing 0.5%SDS), followed by magnetic stirring at 100 rpm at the predesigned temperature. At predetermined time intervals, release media was collected and replaced with an equal amount of fresh media. The released drug was quantified using the HPLC method as mentioned above.

(2) $$\text{Cumulative release}=(\frac{C_{i}*70+C_{i-1}*1+\ldots +C_{1}*1}{C_{F7-\mathrm{TPT}-\mathrm{TSL}}*2})100\%$$

The cumulative release rate (%) was obtained according to Equation (2), where *C_i_* is the measured concentration and (C_F7-TPT-TSL_*2) is the amount of drug in the liposome.

### 2.9. Cell subculture

Human lung large cell carcinoma NCI-H460 (H460) was kindly donated by Prof. Zhi Yao (Tianjin Medical University, Tianjin, China) and was acquired from Shanghai Institutes for Biological Sciences, Chinese Academy of Sciences (Shanghai, China). NCI-H460 cells were cultured in RPMI 1640 medium with 10% (v/v) fetal bovine serum (FBS) and 1% penicillin–streptomycin (100 units/mL, 100 μg/mL) at 37 °C in a humidified atmosphere of 5% CO_2_. Human breast adenocarcinoma cells (MCF-7) purchased from the Cell Resource Center of IBMS (Beijing, China) were maintained in culture medium consisting of DMEM supplemented with 10% (v/v) FBS and 1% penicillin–streptomycin (100 units/mL, 100 μg/mL) at 37 °C in a humidified atmosphere of 5% CO_2_.

### 2.10. Cellular uptake studies

H460 cells (5 × 10^4^ cells/well) were seeded in a six-well plate for 24 h and then treated with free Cou6-DOX solution and CD-TSL (Cou6 and doxorubicin co-loaded thermosensitive liposome). After incubation for 4 h, the media were changed. The cells were washed by PBS three times and then fixed by incubating with 1 mL of 4% paraformaldehyde for 20 min. The cells were then washed by PBS for three times to remove excess agents. For nuclei staining, 1 mL of DAPI (4,6-diamidino-2-phenylindole, 1 μg/mL) solution was added into cells and incubated for 5 min. After the incubation, the cells were softly washed three times to remove excessive DAPI. At last, cells were imaged under inverted fluorescence microscopy (Nikon ECLIPSE Ti). The fluorescence images were taken under a ×10 objective.

### 2.11. Apoptosis assay

Since F7 is a novel compound, no study has published on the ability of F7 compounds and F7 nano preparations to inhibit tumor growth, we first investigated the ability of F7 and F7-TSL to promote apoptosis of tumor cells. We used the Annexin V-FITC kit (Tianjin Sungene Biotech Co., Ltd., Tianjin, China) to detect apoptosis. MCF-7 cells (5 × 10^4^ cells/well) were seeded into six-well plates for 24 h and then were treated with F7 and F7-TSL (0.07, 0.2, 0.7, and 2 μM) for 24 h. Adherent cells were trypsinized and resuspended. After resuspended, cells were added by 5 μL annexin V-FITC and 5 μL propidium iodide (PI) and then incubated for 15 min at room temperature in the dark. The samples were analyzed with a BD FACS Canto II flow cytometer (Becton Dickinson, Franklin Lakes, NJ). Flow cytometry analysis was performed with untreated cells as a control.

### 2.12. Cytotoxicity assay

H460 cells (5 × 10^4^ cells/well) were seeded in a 96-well plate for 24 h. The cells were treated with increasing concentrations F7 (0.004, 0.02, 0.1, 0.5, and 2.5 μmol/L) and TPT (0.004, 0.02, 0.1, 0.5, and 2.5 μmol/L) alone or combination, and heated immediately by immersing the cell-seeded plate in a water bath at 41 °C for 30 min. The cells were subsequently incubated at 37 °C for 47.5 h and their viability was evaluated using an assay with 3-(4,5-dimethyl-2-thiazolyl)-2,5-diphenyl-2H-tetrazolium bromide (MTT). Also, for the normal temperature group, the cell plate after dosing was directly transferred to the incubator for 48 hours and then measured.

### 2.13. Antitumor efficacy *in vivo*

Female nu/nu nude mice (weighing 18–22 g) were purchased from Vital River Laboratories (Beijing, China). A xenograft tumor model was produced via a subcutaneous injection of MCF-7 cells. All procedures involving animal housing and treatment were approved by the Animal Care and Use Ethics Committee of the Academy of Military Medical Sciences.

Fifty-four female nude mice (MCF-7) were randomly divided into the control group (saline sham), F7 solution group, TPT solution group, F7-TPT solution, F7-TPT solution/H group, F7-TSL group, TPT-TSL group, F7-TPT-TSL group, and F7-TPT-TSL/HT group (*n* = 6).

The administration method was tail vein injection, and the heated group was contacted with a 42.5 °C copper column to heat the tumor site for 30 min. The treatment interval was four days and the number of treatments was six times. The above-mentioned preparations were administered to nude mice at a concentration of 100 μg/mL, and the dose was 1 mg/kg (the combined drug group dose is F7 0.5 mg/kg + TPT 0.5 mg/kg). Tumor volume was continuously monitored during dosing, and body weight changes were recorded.

Two days after the end of treatment, the above nude mice were humanely euthanized, the tumor tissues were excised and weighed, and the tumor inhibition rate was calculated.

During the experiment, the weight of mice was affected by many factors, including the growth of mice, the increase of tumor, and the systemic toxicity of drugs. It is not very accurate to just use the weight change of mice to evaluate the drug toxicity. The relative weight change rate was used to evaluate the drug toxicity, and its calculation formula is as follows:

(3) $$\text{Relative weight change rate }(\%)=\frac{\text{body weight }22\text{ d }-\text{ body weight }0\text{ d }-\text{ tumor weight}}{\text{body weight }0\;d}$$

Relative weight change rate was obtained according to Equation (3), where body weight 22 d are body weight of mice treated for 22 days and body weight 0 d are initial body weight of mice.

(4) $$\text{Tumor volume}=\frac{\mathrm{lengh}\times {\mathrm{width}}^{2}}{2}$$

Tumor volume was obtained according to Equation (4), where length and width are the maximum and minimum diameters of a tumor, respectively

(5) $$\text{Tumor inhibition rate }(\%)=(1-\frac{M_{\text{therapy group}}}{M_{\text{control group}}})100\%$$

Tumor inhibition rate (%) was obtained according to Equation (5), where *M*_therapy group_ indicates mean tumor mass of the treatment group and *M*_control group_ indicates the mean tumor mass of the control group.

## 3. Results

### 3.1. Preparation and characterization of F7-TSL

**Figure 1.**
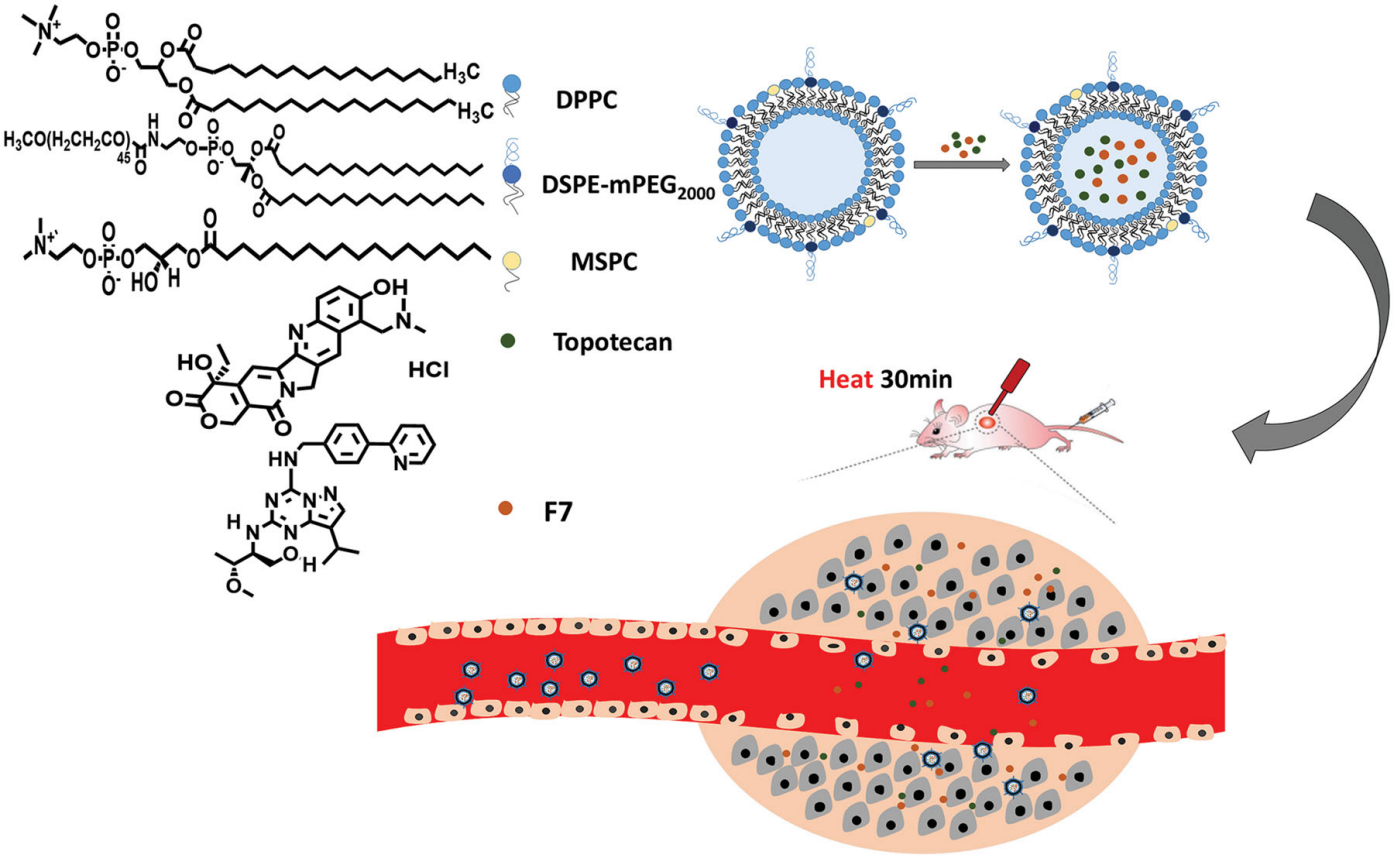
Schematic illustration of the fabrication process and structure of F7-TPT-TSL and its applications in chemotherapy combined with hyperthermia.

Figure 1 shows a schematic of F7-TPT-TSL loaded with F7 and TPT. F7-TPT-TSL was prepared by the pH gradient method based on the nature of the drug itself. F7 has high solubility under acidic conditions and is nearly insoluble under neutral conditions. TPT can maintain an active lactone structure in an acidic environment, and a pH gradient is formed by adjusting the pH of the internal and external aqueous phases. The drug enters the internal aqueous phase of the liposome in the molecular form and is stabilized in the ionic form. Various liposomes loaded with F7 and TPT were prepared, and their particle size, zeta potential, and encapsulation efficiency (EE%) were determined, as summarized in Table 1.

**Table 1.** Physicochemical properties of different liposome preparations.

Code	Encapsulation efficiency (%)		Particle size (nm)	PDI	Potential (mV)
	F7	TPT			
Blank TSL	–	–	89.86 ± 3.34	0.173 ± 0.016	–8.62 ± 1.01
F7-TSL	99.97 ± 2.27	–	111.63 ± 8.81	0.201 ± 0.020	–15.48 ± 6.20
TPT-TSL	–	98.73 ± 0.39	105.64 ± 8.68	0.200 ± 0.020	–16.91 ± 3.32
F7-TPT-TSL	97.42 ± 0.74	95.15 ± 4.23	103.00 ± 9.84	0.194 ± 0.015	–14.28 ± 4.02

**Figure 2.**
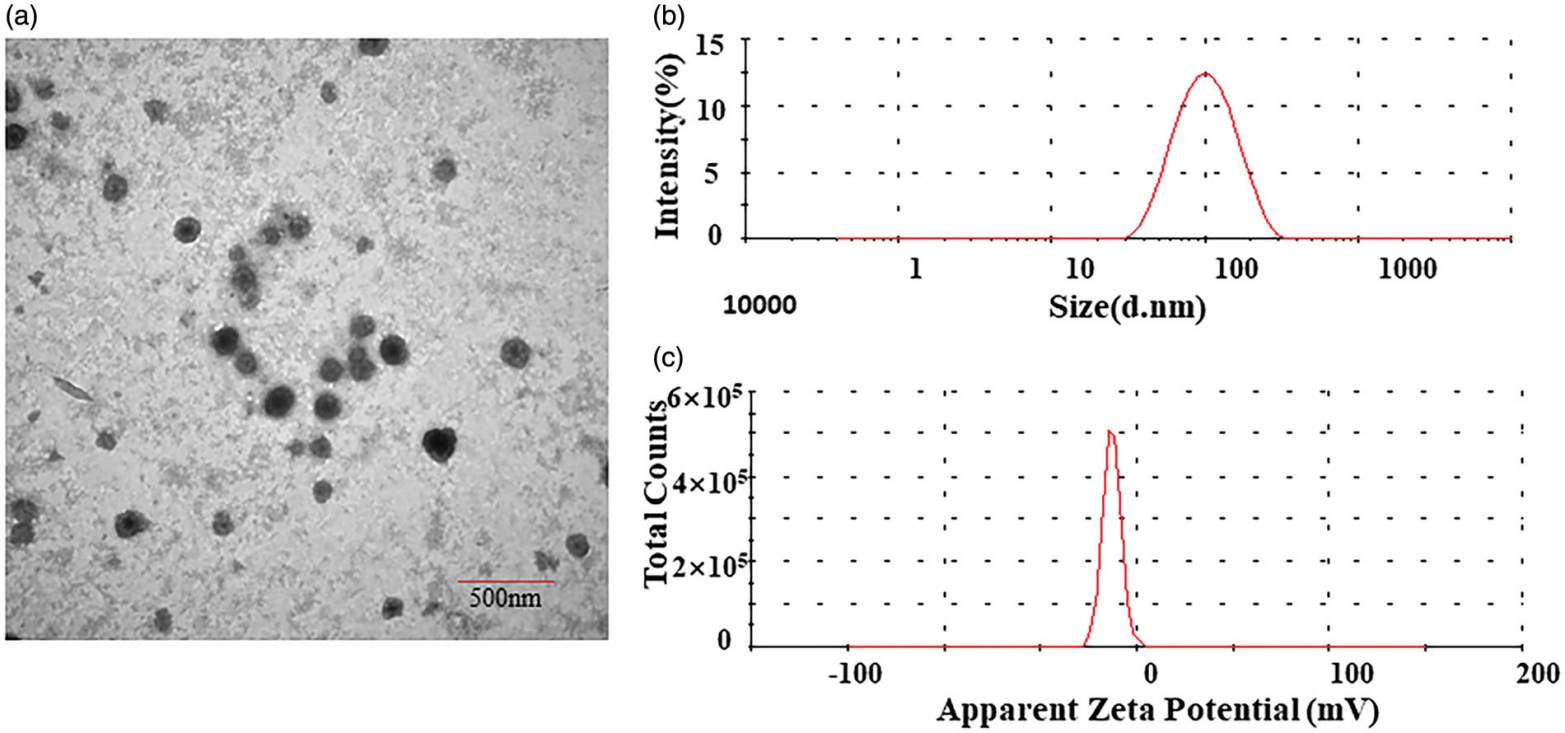
TEM image (a), particle size distribution (b), and potential distribution (c) of F7-TPT-TSL.

By using a transmembrane gradient of pH, we achieved efficient loading of F7 and TPT in the aqueous phase of the liposome. The TSL showed an encapsulation efficiency of more than 95%, a particle diameter of approximately 100 nm, and narrow particle size distribution (PDI < 0.3), showing a good distribution. The particle size distribution graph and potential distribution graph of F7-TPT-TSL are shown in Figure 2.

### 3.2. Morphology analysis

The morphology of F7-TPT-TSL was observed by TEM. As shown in Figure 2, the liposome was spherical or oval, and the morphology was consistent with the assumed result.

### 3.3. Liposomal phase transition temperature analysis

**Figure 3.**
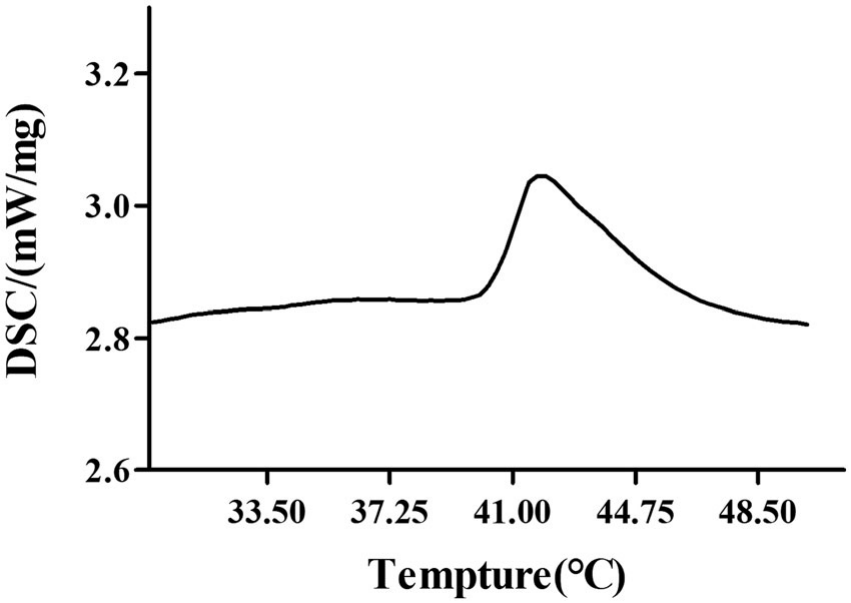
Differential scanning calorimetry phase transition temperature map.

The phase transition temperature of the main membrane DPPC prepared in this study was 41 °C, and the interaction of different ratios of phospholipids enabled the preparation of liposomes with different phase transition temperatures. Blank liposomes were prepared and showed a phase transition temperature of 40.3 °C, which is slightly lower than that of DPPC (Figure 3). This temperature is suitable for inducing hypothermia in human tumors.

### 3.4. Thermal release analysis

**Figure 4.**
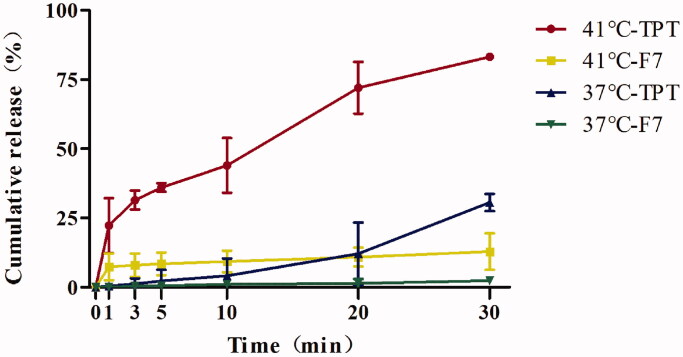
Temperature-triggered release behaviors of F7-TPT-TSL at 41 °C and 37 °C (*n* = 3).

To verify the thermal sensitivity of F7-TPT-TSL, we evaluated the drug release profile. We performed three release experiments. As shown in Figure 4, after dialyzing co-loaded F7-TPT-TSL at 41 °C, the release of TPT showed a significant thermal response. In the first 5 min of heating, the average release of TPT reached 36%; after heating for 30 min, the average release was 83%. However, the release of the F7 showed an average rate of only 11% after heating for 30 min. The thermosensitive release was verified *in vitro*, showing a good release profile of the water-soluble drug TPT. The reason for the low release of F7 was the structural characteristics of the drug itself. F7 is an amphiphilic compound that is fat-soluble. Therefore, even if the membrane morphology of the TSL changes from a colloidal state to a liquid crystalline state, the lipid bilayer membrane remains intact. When F7 penetrates the phospholipid bilayer, it sticks to the middle of the bilayer membrane, affecting drug release detection and causing the *in vitro* release data to deviate from the ideal state. The release profile *in vitro* showed that when the TSL was at the phase transition temperature, the TPT release was accelerated, which did not significantly affect F7. At 37 °C, 30.60% of TPT and 2.34% of F7 were released. The transition of the liposome membrane from the colloidal state to the liquid crystal state was not instantaneous; first, a pre-phase transformation occurred at a temperature of approximately 5 °C lower than the main phase. Between the pre-phase transition and main phase transition, the membrane phases became separated and the membrane structure became hollow, releasing the encapsulated drug at a slow rate.

### 3.5. Stability analysis

**Figure 5.**
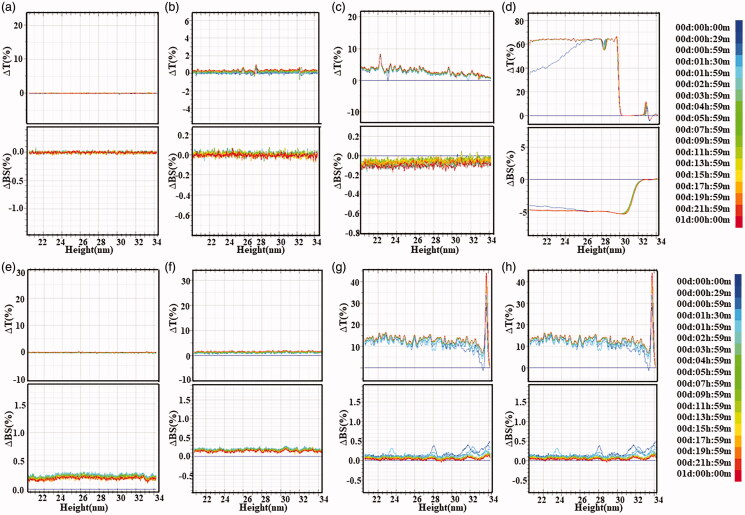
Transmission and backscattering profiles of liposome preparation by using a Turbiscan Lab@ Expert. The stability image (a) TSL, (b) F7-TPT-TSL, (c) F7 solution, (d) TPT solution diluted with water for 24 h at 25 °C, (e) TSL, (f) F7-TPT-TSL, (g) F7 solution, and (h) TPT solution diluted with serum for 24 h at 37 °C. Data are reported as a function of time and sample height (20–34 mm).

As shown in Figure 5, the physical stability of blank-TSL and F7-TPT-TSL was analyzed using a stability meter. After storage for 1 month at 4 °C, the blank liposomes and co-loaded liposomes were stable in water and serum, indicating that the liposome preparations had high physical stability and showed no aggregation or sedimentation. We also tested the stability of the F7 and TPT powder. As a result, the stability of TPT fluctuated widely, likely because of the lactone ring in the structure. Therefore, F7 and TPT can be effectively encapsulated in liposomes.

### 3.6. Cellular uptake assay

**Figure 6.**
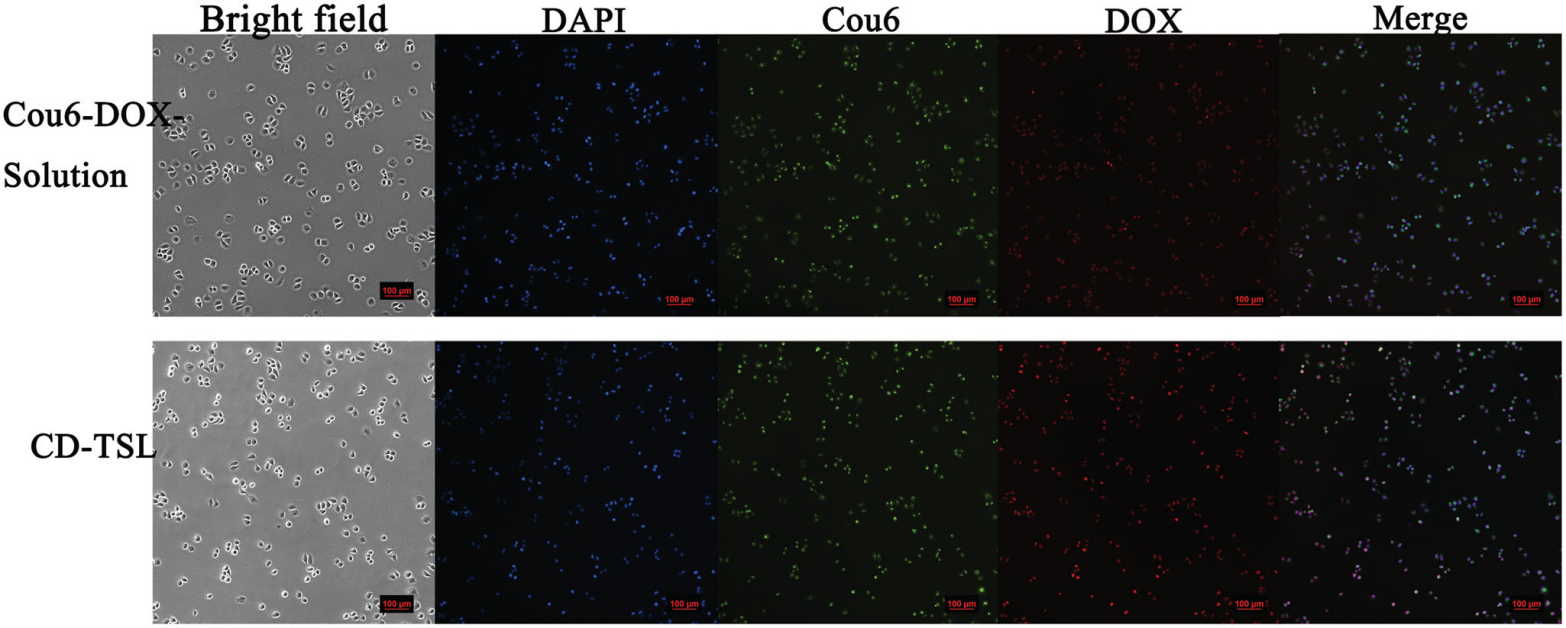
Cellular uptake of Cou6-DOX-solution and CD-TSL by H460 cells after 4 h incubation at 37 °C. Images of bright field, nuclei stained with DAPI (blue), 6-coumarin (green), DOX (red), and merged. Scale bar: 100 μm.

The cell-penetrating efficiency of TSL was further evaluated by inverted fluorescence microscopy. To clarify the internalization of the liposomes, the cell nuclei were stained with DAPI (blue) and Cou6-DOX-solution which shows green fluorescence (Cou-6) and red fluorescence (DOX). CD-TSL shows red fluorescence and green fluorescence. As shown in Figure 6, CD-TSL had stronger red fluorescence intensity than Cou6-DOX-solution had, indicating that the phospholipid membrane facilitates low fat-soluble drug entry to cells through fusion effects, while water-soluble drugs have poor ability to enter cells. For Cou-6 distributed in the TSL membrane, images of the green fluorescence of CD-TSL showed that TSL could enter the cells via membrane fusion.

### 3.7. Cell assay *in vitro*

**Figure 7.**
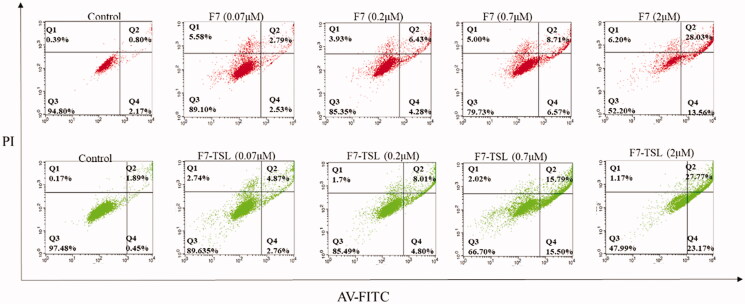
Apoptosis analysis of MCF-7 cells induced by F7 solution and F7-TSL for 24 h.

Flow cytometry studies were performed to calculate the cytotoxic potential of different F7 formulations in terms of apoptosis and necrosis (Figure 7). F7 solution at 0.07, 0.2, 0.7, and 2 μM showed apoptosis rates of 5.32%, 10.71%, 15.28%, and 41.59%, respectively, and F7-TSL at 0.07, 0.2, 0.7, and 2 μM showed apoptosis rates of 7.63%, 12.81%, 17.67%, and 50.94%, respectively (Figure 7). With increasing drug concentrations, cell survival gradually decreased whereas apoptosis and necrosis gradually increased, showing concentration-dependent effects.

**Figure 8.**
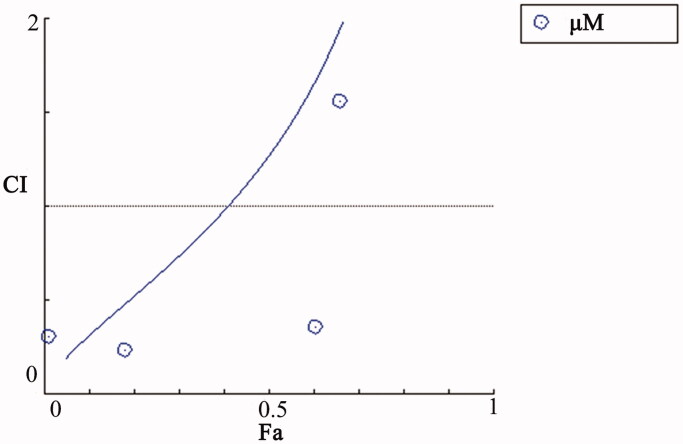
Interaction between F7 and TPT affects H460 cell growth. The graph shows CI values after treatment. CI < 1 represents a synergistic effect and CI > 1 indicates a less than additive effect of combined treatment. CI = 1 indicates that the effect is additive. CI values for the combination of F7 and TPT were calculated using CompuSyn software.

**Figure 9.**
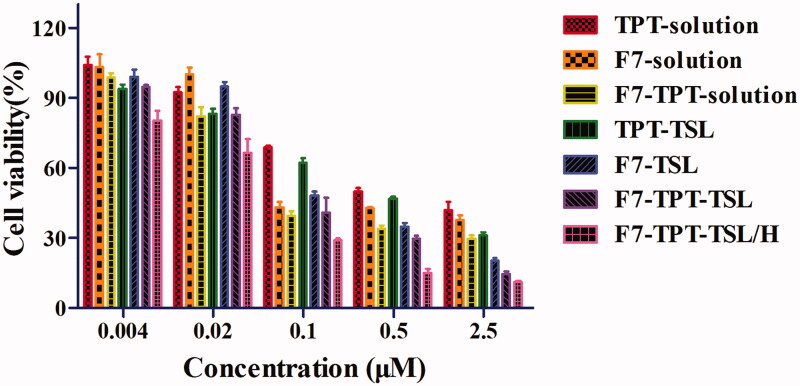
Cell viability of different preparations and concentrations. F7-TPT-TSL with TPT + F7 solution (*p* < .001); F7 – TPT-TSL/H with TPT + F7 solution (*p* < .001); and F7-TPT-TSL/H with F7 + TPT-TSL (*p* < .001).

Cell survival of normal drugs and different liposome preparations after normal treatment and auxiliary heat treatment was determined using the MTT assay. The cytotoxicity of different concentrations of TPT solution, F7-solution, F7 + TPT solution, TPT-TSL, F7-TSL, F7-TPT-TSL, and F7-TPT-TSL/H was investigated to detect dose-dependent anticancer activity. The half-maximal inhibitory concentration (IC_50_) of each formulation is listed in Table 2. As shown in Figure 8, the drug combination (F7 and TPT solution) exhibited a synergistic effect (CI < 1) (total dose of 0.008–0.2 μΜ). As the concentration increased, all formulations showed significantly increased cytotoxicity. The combination drug group showed stronger cytotoxic effects than F7 or TPT monotherapy. Additionally, higher toxicity was observed when the drug was encapsulated in the liposomes (*p* < .05), for both the single drug effect or via co-stimulation of F7 and TPT, particularly after heating (*p* < .05). Importantly, cell viability in the heated group was significantly different compared to the normal liposome group (*p* < .001) (Figure 9). This is because, under normal circumstances, liposomes are more likely than small molecule drugs to enter cells; heating at 41 °C increased the permeability of the cell membrane, making the cells more likely to take up liposomes. A high temperature accelerated the accumulation of F7 and TPT in the cells, and then acted on different targets to kill the cells, eventually achieving a synergistic effect.

**Table 2.** IC_50_ values of different formulations.

Different formulations	TPT solution	F7 solution	F7 + TPT solution	TPT-TSL	F7-TSL	F7-TPT-TSL	F7-TPT-TSL/H
IC_50_ (μΜ)	0.8184	0.4426	0.3052	0.3951	0.1828	0.2154	0.07524

### 3.8. Inhibition of tumor growth *in vivo*

**Figure 10.**
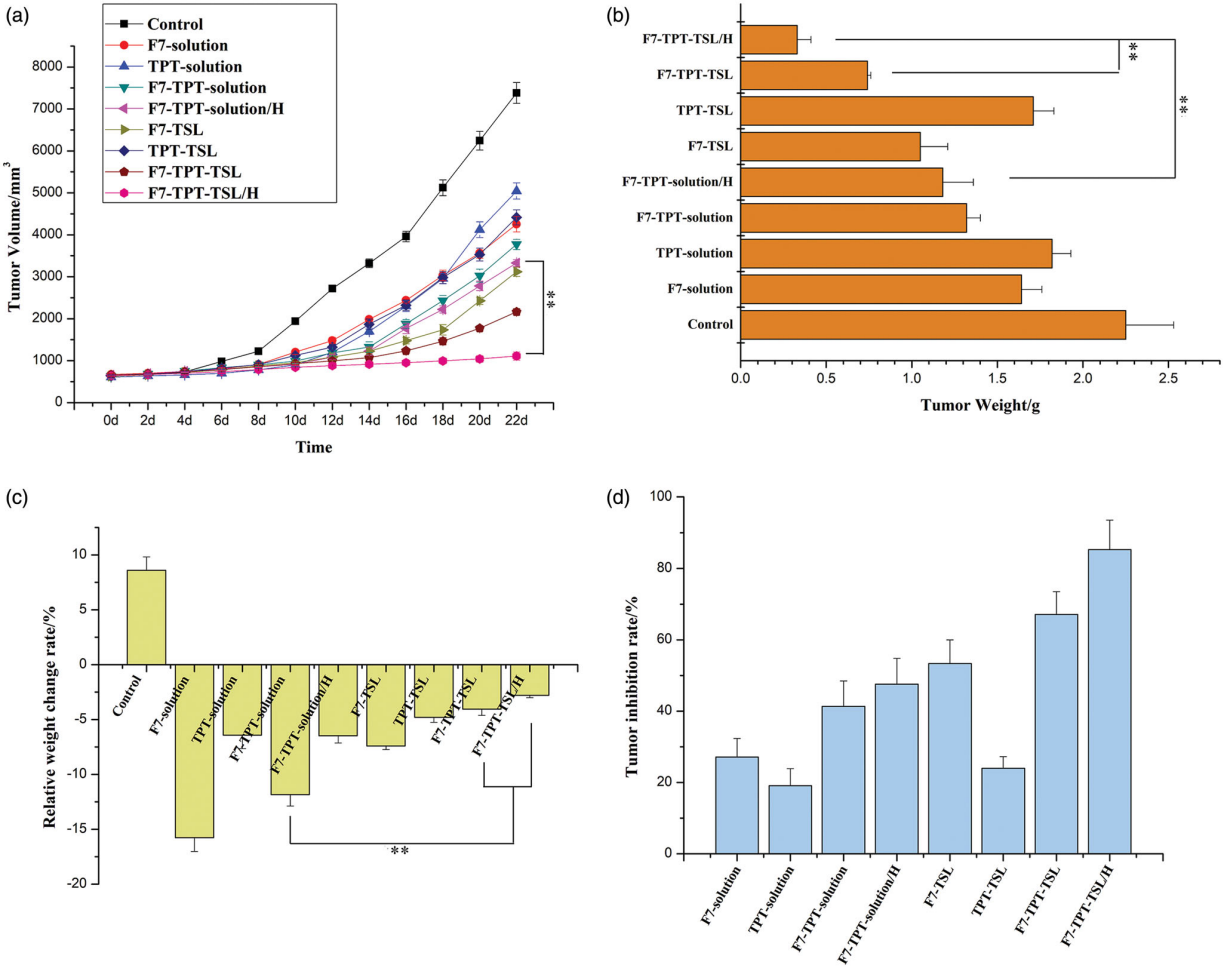
(a) Anticancer efficacy in MCF-7 xenografts in female nude mice after treatment with varying formulations; (b) weights of dissected tumors at the end of treatment; (c) relative weight change of varying formulations in tumor-bearing nude mice; (d) tumor inhibition rate of varying formulations in tumor-bearing nude mice. **p < .01 (*n* = 6).

We recorded changes in the mouse body weight and tumor size. As shown in Figure 10(a), PBS did not inhibit tumor growth and rapidly achieved maximum tumor volume at the end of day 22. Delivery of free drugs (1 mg/kg) did control the growth of tumors to an extent however inefficient in completely controlling its proliferation. The F7-TPT-TSL/H exhibited relatively better tumor growth inhibition by comparison to free F7-TPT-solution. As expected, F7-TPT-TSL/H showed significant (*p* < .01) inhibition of tumor growth compared with F7-solution and TPT-solution. This could be due to the synergistic inhibitory effect of F7 and TPT on the tumor proliferation. The tumor mass was excised and the weight of individual tumors is presented in Figure 10(b). Consistent with the tumor volume, F7-TPT-TSL/H showed a significantly (*p* < .01) smaller tumor size compared to that of either F7-TPT-solution or F7-TPT-TSL. The calculated rate of relative weight change is shown in Figure 1(c) and Table 3. The weight of mice in the treatment group showed a negative increase, and F7-solution was the most obvious. The simple drug combination (F7-TPT-solution) did not change this situation. However, F7-TPT-TSL seems to alleviate toxic and side effects (*p* < .01). Of course, this requires more in-depth research to confirm that the combination of drug TSLs can reduce systemic toxicity. Figure 10(d) shows the tumor weight and tumor inhibition rate after 22 days of treatment in mice. Overall, the tumor inhibition rate of the liposome group is greater than that of the corresponding free drug, specifically, F7-TPT-TSL/H > F7-TPT-TSL > F7-TSL > F7-TPT-solution/H > F7-TPT-solution > F7-solution > TPT-TSL > TPT-solution. The remarkable tumor efficacy was due to the combination of thermal-liposome carrier and hypothermia which further increased the chemotherapeutic efficacy of drug combination. Encapsulation of the two drugs in the liposome significantly improved the efficacy and avoided the strong systemic toxicity caused by a single use of the F7 solution. Therefore, by reducing systemic toxicity or the anti-tumor growth ability, the F7-TPT-TSL/H group achieved a more prominent effect than the other groups.

**Table 3.** Relative weight change of different formulations.

Different formulations	Control	F7-solution	TPT-solution	F7-TPT-solution	F7-TPT-solution/H	F7-TSL	TPT-TSL	F7-TPT-TSL	F7-TPT-TSL/H
Tumor weight (g)	2.25 ± 0.28	1.64 ± 0.12	1.82 ± 0.11	1.32 ± 0.08	1.18 ± 0.18	1.05 ± 0.16	1.71 ± 0.12	0.74 ± 0.02	0.33 ± 0.08
Body weight (g)/0 d	20.82 ± 0.56	20.88 ± 0.96	20.86 ± 0.74	20.78 ± 0.22	20.68 ± 0.15	20.92 ± 0.45	21.04 ± 0.33	20.78 ± 0.42	20.85 ± 0.53
Body weight (g)/22 d	24.86 ± 1.61	19.23 ± 1.12	21.34 ± 1.14	19.64 ± 1.52	20.52 ± 0.86	20.42 ± 0.76	21.74 ± 0.84	20.68 ± 0.56	20.60 ± 0.88
Relative weight change (%)	8.60 ± 1.23	–15.76 ± 1.26	–6.42 ± 1.22	–11.84 ± 1.04	–6.48 ± 0.65	–7.41 ± 0.32	–4.80 ± 0.46	–4.04 ± 0.57	–2.78 ± 0.22

The most characteristic features of tumors are invasive and rapid growth. When the tumor diameter reaches 2 mm^3^, vasculature-like angiogenesis of the mutated cancer cells provides nutrition to the tumor; the cancer cells are loosely distributed and disordered, and the vascular wall gap is 380–780 nm (Ballet, 1990; Liechty & Peppas, 2012; Sun, 2016). This appropriate gap size allows the liposome formulation to permeate the tumor vessel wall and remain in tumor tissue sites, and heating triggers the rapid release of the drug to achieve better anti-tumor effects and reduce systemic toxicity (Zhang et al., 2016). F7-TPT-TSL showed an appropriate particle size of approximately 100 nm, enabling the PEGylated lipid to evade phagocytosis of the reticuloendothelial system during blood circulation and accumulate at tumor sites via the enhanced permeability and retention effect. When the TSL reached the tumor tissue site, a high temperature of 41 °C was applied to increase cell membrane permeability and drug accessibility at the target cells. The TSL showed a phase change, and both drugs were rapidly released to act on the tumor cells to inhibit growth and avoid resistance caused by a single drug. As shown in Figure 10, F7-TPT-TSL/H showed the strongest ability to inhibit tumor growth.

## 4. Discussion

Liposomes are used as drug carriers for tumor treatment because of their biocompatibility, biodegradability, particle size, hydrophobic and hydraulic characteristics, flexible structure, non-toxicity, and non-immunogenicity (Haeri et al., 2016). We prepared F7 and TPT co-loaded TSL with membranes composed of DPPC, DSPE-mPEG2000, and MSPC. The exact temperature and width of the phase transition are determined by the lipid composition (de Matos et al., 2018). The TSL was prepared from a synthetic phospholipid, and the phase transition temperature of the main material, DPPC, was 41 °C. As the three phospholipid materials were mixed in a specific ratio, the resulting liposomes showed a phase transition temperature of 40.3 °C (Figure 3). The addition of DSPE-mPEG_2000_ can help liposomes escape phagocytosis by the reticuloendothelial system in the blood circulation and prolong the circulation time of liposomes *in vivo*. Minimal MSPC was added as a catalyst to destroy the lipid bilayer.

TPT and F7 were co-loaded into the liposomes. The drugs entered the internal aqueous phase of the liposomes in molecular form and were stable in the form of ions. By measuring the levels of both drugs by high-performance liquid chromatography, the amounts of both drugs in the co-loaded liposomes can be precisely controlled. As the encapsulation efficiency of both drugs was greater than 95%, it was unnecessary to remove the free drug from the external aqueous phase. The size of liposomal particles can significantly affect their *in vivo* behavior, and the carrier (liposome) releases the drugs upon external stimulation (Ott et al., 2017).

Synchronous release of both drugs can optimize synergy. In this study, the *in vitro* release data deviated from the ideal state at 41 °C. When the TSLs were at the phase transition temperature, TPT release was accelerated, whereas that of F7 was not significantly affected (Figure 4). However, simple *in vitro* release does not simulate the pattern of drugs in the body. Passive diffusion through lipid membranes is a common mode of transport of fat-soluble drugs. Besides, liposomes can enter cells by endocytosis, membrane fusion, and other mechanisms. Thus, encapsulated drugs can be delivered to the cells simultaneously. Based on the *in vivo* environment and methods of liposome entry into cells, the F7-TPT-TSL formulation was located in the tumor tissue and the loaded drugs were synchronized. The MTT assay validated the synergistic effects of the drugs (Figure 8).

The fate of liposomes *in vivo* was further investigated based on the differences between the *in vitro* and *in vivo* environments. The biological basis of hyperthermia is that with increasing temperature, cell membrane fluidity, and cell sensitivity both increased. The combination of hyperthermia and chemotherapy has been widely studied. Hyperthermia increases blood flow and vascular permeability in the tumor, enabling liposomes to widely infiltrate the tumor, thus improving the local drug concentration (Li et al., 2016). In this study, the heating group was contacted by a 42.5 °C copper column to heat the tumor site. The results showed that the F7-TPT-TSL/H group achieved prominent effects regardless of systemic toxicity or anti-tumor growth ability (Figure 10). Therefore, co-loaded F7-TPT-TSL was the best preparation for inhibiting tumor growth.

Co-loaded liposomes have been shown to have enhanced therapeutic potential for cancer. Both cytotoxicities *in vitro* and antitumor activity studies *in vivo* have demonstrated the superior efficacy of co-loaded liposomes compared to their solution mixtures or liposomes loaded with a single drug. These results confirm that co-loaded TSLs can enhance anti-tumor therapeutic effects by controlling the ratio of effective drugs in target tissues.

## 5. Conclusions

In this study, the constructed delivery system F7-TPT-TSL showed good physico-chemical features such as uniform particle size, near zero zeta potential, really proud entrapment efficiency, obvious thermo-sensitive characteristics, and excellent *in vitro* stability. More importantly, the vesicles exhibited strong tumor inhibitory activities when they were combined with hyperthermia, both *in vitro* and *in vivo*. Based on the bio-distribution of TSL, we have made full use of the synergistic effects of TPT and F7 in tumor suppression, and the restriction of their toxicity. The promising results warrant future studies which involve improve *in vivo* study including survival analysis and tissue distribution of F7-TPT-TSL, as well as develop more potential drug combination encapsulated in TSL to achieving much greater therapeutic effects. And for many novel compounds similar to F7 (high efficiency but too high toxicity), TSL may totally change their *in vivo* bio-distribution and decrease systemic toxicity, expanding their applications in chemotherapy.
